# Converting of CO_2_ into low-molecular-weight organic compounds with the TiO_2_/ZrO_2_ composites under solar irradiation

**DOI:** 10.1038/s41598-017-14683-4

**Published:** 2017-10-31

**Authors:** Ichiro Moriya

**Affiliations:** South wing 101, Maebara-nishi 3-6-3, Funabashi, Chiba, Japan

## Abstract

The preparation of a specially modified titanium dioxide/zirconium oxide (TiO_2_/ZrO_2_) composite and its subsequent application using a unique method are described. Specifically, after the whole surface of the composite was covered with a very thin layer of water, solar light was irradiated onto it. This method is unique because the reduction of CO_2_ was performed in air (gas phase). The light source was real solar light. In this study, novel composites comprising nanometre-sized TiO_2_ and micrometre-sized zirconium oxide (ZrO_2_) increased the amount of reduced CO_2_. And, suitable weight ratio of TiO_2_/ZrO_2_ was 6/4-5/5. Thin water layer on the composite offered catalytic-reaction medium, and, catalytic-reaction cite existed at interface of TiO_2_ and ZrO_2_ particles, and, this reaction was catalytic reaction enhanced by photocatalytic effect. A large amount of reduced products (maximum: approximately 300 μmol/(g·300 s) of formaldehyde and methanol)was obtained under only 300 s of irradiation of solar light.

## Introduction

The CO_2_ concentration in the atmosphere is gradually increasing. Temperature of atmosphere is rising continuously^1^. Research into methods for converting CO_2_ into low-molecular-weight organic compounds is extremely important because this research can not only reduce the concentration of CO_2_ in the atmosphere but also can convert the CO_2_ into useful organic compounds. Reported methods for reducing CO_2_ include the use of photo-catalysts or photo-electrodes^2,3^. Sato *et al*. succeeded in converting CO_2_ into organic compound (formic acid (HCOOH)) under irradiation of solar simulated light^4^. This report was probably most advanced one in this field on that time (2011). But, the reduced product was small(approximately 1 μmole/4 h cm^2^ under irradiation intensity 1 sun (AM 1.5)). In recent times, many reports in regard to converting CO_2_ into organic compounds with special photo-catalysts under solar light or visible light irradiation have been proposed^5–9^. However, their reduced products were small, too. Moreover, there are excellent review literatures^10–12^. With regarded to large reaction products, Zhao *et al*. reported that total organic carbon (TOC) yield of N-TNT(Nitrogen-doped TiO_2_ nanotube) 500 (post-heating 500 °C) was 1453.0 μmol/g-cat. in 100 ml 0.1 N NaOH aqueous solution with a visible-light of 500 W tungsten-halogen lamp^13^.

And, Wang *et al*. reported Pt-TiO_2_ nanostructured films via versatile gas-phase deposition method (unique one-dimensional structure of TiO_2_ single crystals coated with ultrafine Pt nanoparticles (0.5–2 nm)) and maximum Methane (CH_4_) yield of 1361 μmol/g-cat./h with 400 W-Xe lamp (accumulated intensity of 19.6 mW/cm^2^ in the effective UV range (250–388 nm))^14^.

On the other hand, in catalytic field, commercial methanol producing process have established, the process is commercially in operation with Cu/ZnO/Al_2_O_3_ or Cu/ZnO/Cr_2_O_3_ catalyst by using of CO_2_-H_2_ system under high temperature and high pressure. CO_2_ and H_2_ are those obtained by another industrial processes.

Millar *et al*. reported importance regarding to interface of particles that constructed catalyst^15,16^. However, on the catalytic fields, there are no ideas of using CO_2_ in the air and of using solar light as energy source.

Therefore, no report in which a very large amount of organic compounds has been generated by reducing CO_2_ with inexpensive catalyst in air under irradiation of solar light at room temperature and atmospheric pressure in both photocatalytic and catalytic fields is founded.

The aim of this research is obtaining large amount of low-molecular- weight organic compounds by the original method under irradiation of real solar light.

In relation to this research, Fujishima *et al*. reported the photo-catalyst effect of TiO_2_^17^. Specifically, irradiation with UV light generates electrons (e^−^) and holes (h^+^) in TiO_2_. The electrons provide the reduction driving force, and the holes provide the oxidation driving force. In 1979, Inoue and Fujishima *et al*. reported the reduction of CO_2_ to formaldehyde (HCHO) and methanol (CH_3_OH) under irradiation with high-energy UV light using a TiO_2_ photocatalyst^18^. Later, Sato *et al*. reported the generation of H_2_ and O_2_ using wet Pt/TiO_2_ under irradiation by light with energy equal to the band-gap energy of TiO_2_ (1980)^19,20^. Moreover, they demonstrated that H_2_ and O_2_ were generated by the splitting of H_2_O added. Author also started this study to obtain desirable result by the photocatalytic reaction.

In this report, following items were studied.

First: Ascertaining whether the thin water layer on the surface of composites is essential.

Second: Consideration of preparing method (Effect of press on preparing method of composites)

Third: Dependence of quantity of reduced products on TiO_2_/ZrO_2_ ratios. (TiO_2_/ZrO_2_ ratios were changed between 10/0 and 0/10).

Forth: Measurements of quantity of reduced products under irradiation of real solar light (at TiO_2_/ZrO_2_ = 1/1).

Fifth: Proving the water layer on the surface of composites.

Sixth: Discussion regarding with macro mechanism (hypothesis) through which the large quantity reduced product can be obtained.

## Experiments

The author devised composites consisting of nanometre-sized TiO_2_ particles and micrometre-sized zirconium oxide (ZrO_2_) particles and applied them using an original method. First, particles or molecules adsorbed onto the composite surface were removed by the elimination of static electricity using an ion blower. Next, after the composites were cooled in a refrigerator for more than 20 h, they were placed into a transparent gas-barrier plastic bag with high-temperature and high-humidity room air.

Cooling in a refrigerator enabled the whole surface of the composite to be covered with a very thin layer of water via the condensation of water vapour in the air to reproduce Sato’s condition (wet condition). The reduction of CO_2_ did not occur until the thin water layer condensed onto the composite’s surface. This method is unique because the reduction of CO_2_ is performed in air (or more precisely, in the air phase via a very thin water layer). Moreover, this method increases the efficiency of the light irradiation because real solar light is directly irradiated onto the composites.

In this study, the TiO_2_/ZrO_2_ composites greatly increased the amount of reduced CO_2_ product. The composite exhibited a particulate morphology. In this report, CO_2_ was reduced under real solar light without the use of platinum as a co-catalyst.

The new photo-catalyst composites are composed of two types of inorganic compounds (a,b) that were combined and pressed together. The first inorganic compound (a) is characterized by nanometre-sized anatase TiO_2_ photo-catalyst particles, and the other inorganic compound (b) is composed of micrometre-sized ZrO_2_ particles. The weight ratio (TiO_2_/ZrO_2_) of the composites is mainly 1:1. The use of such a large amount of additive (ZrO_2_) is unique. After (a) and (b) were pressed to form the composites (c), the composites (c) were scattered onto an electric conducting material such as a copper plate. The copper plate can facilitate the transfer of electrons and holes from one composite to the other. Then, the composites and Cu plate were held in a glass laboratory dish (the glass laboratory dish containing the composites and Cu plate is hereafter referred to as the test unit).

**Figure 1:**
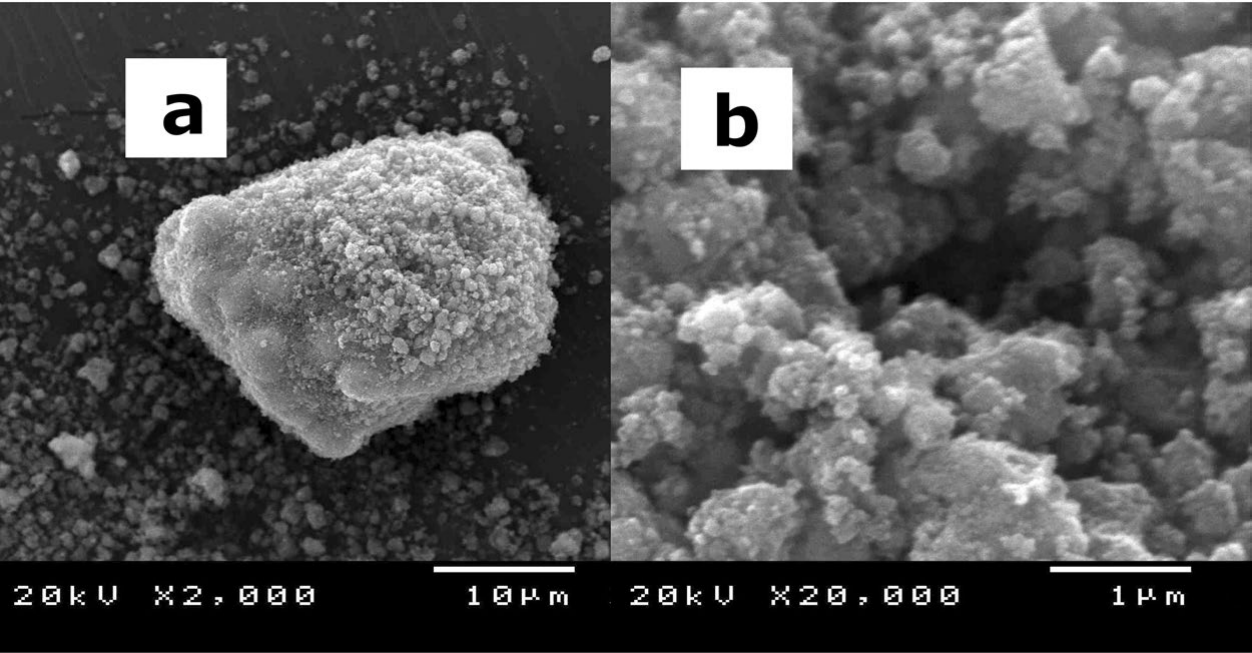
Magnification SEM image of the composite. (**a**) Low-magnification SEM image of the composite. This image is a low-magnification (2000×) micrograph of typical composite particle. (**b**) High-magnification SEM image of the composite. This image is a high-magnification (20,000×) image of the surface shown in “a” of Fig. 1.

Figure 1 shows SEM image of the composite with water vapour condensed on its surface and with the thin water layer evaporated away (SEM: JAPAN ELECTRONIC Co. Ltd., JSM-5310LV). Image “a” shows that numerous nanometre-sized TiO_2_ particles are present on the top layer of the composite, and the core of the composite is composed of micrometre-sized ZrO_2_ particle. Image “b” shows a higher-magnification (20,000×) SEM image of the surface of the composites shown in image “a” of Fig. 1. Pores of various sizes are present in the nanometre-sized TiO_2_ layer. The CO_2_ in the air can penetrate the pores and reach the water layer existing at the surface of the ZrO_2_ particles.

**Figure 2:**
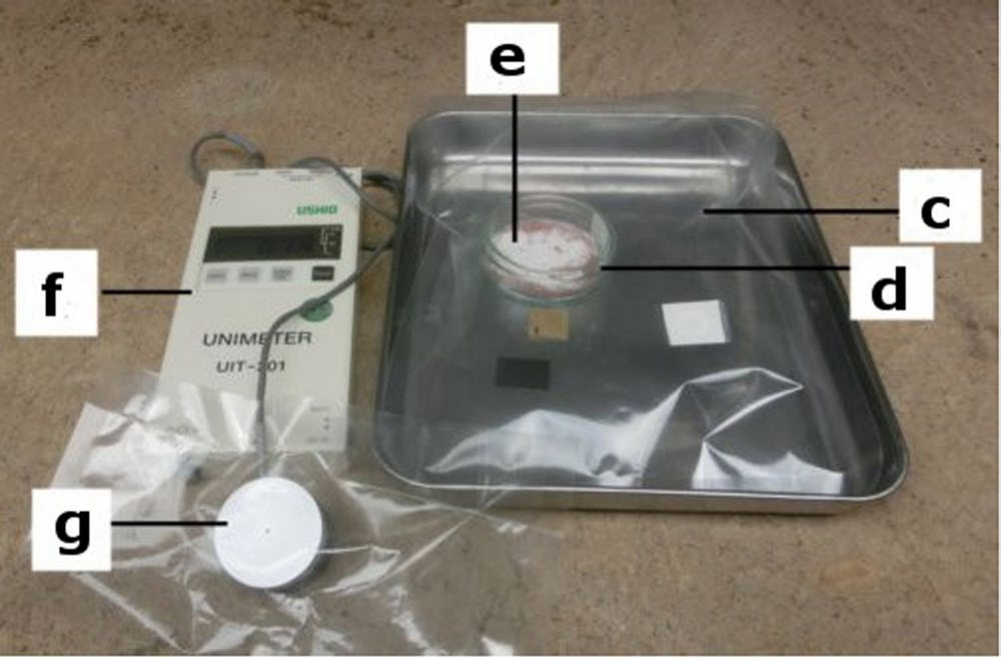
Photograph taken during the experiment. (**c**) Gas-barrier plastic bag; (**d**) test unit; (**e**) composites (TiO_2_:ZrO_2_ = 1:1); (**f**) solar-energy-detecting instrument (UNIMETER); (**g**) solar-energy-detecting sensor. The composites in the test unit were irradiated through the gas-barrier plastic bag. The only light source was real solar light.

Figure 2 shows a photograph of the experiment under irradiation by real solar light. Experiments were performed during the Japanese summer season, which provides high temperatures and high humidity. During irradiation, the test unit was placed into a transparent gas-barrier plastic bag with 1000 ml of air. The bag transfers solar light and contains the produced organic compounds. The bag allows both the concentration of organic compounds and the inner volume to be measured after solar-light irradiation. After irradiation, the concentration of formaldehyde and methanol in the bag was measured using gas-detecting tubes.

A gas-barrier plastic bag through which CO_2_ molecules can penetrate but not oxygen (O_2_) or nitrogen molecules (N_2_) was used. When the amount of CO_2_ in the bag is decreased by the reduction of CO_2_, the CO_2_ in the air outside of the bag diffuses into the bag because of the concentration gradient. Therefore, the concentration (in ppm) of the total reduced products exceeding the CO_2_ concentration in the atmosphere (approximately 400 ppm) is not contradictory.

The experiments were conducted in Funabashi in the Chiba prefecture of Japan (lat. 35° 70′ N and long. 140° 02′ E).

Additionally, the author performed several weight measurements 5–8 min after removing the test unit from the refrigerator. The weight of the test unit increased by 70–110 mg because of the condensation of water vapour (ambient air conditions: 30.9 °C in temperature and 65% in humidity −30.0 °C in temperature and 90% in humidity).

## Results

In the subsequent description, the term “dry conditions” refers to experiments in which the test unit was not cooled in a refrigerator, whereas the term “wet conditions” refers to experiments in which the test unit was cooled in a refrigerator, resulting in the formation of a thin water layer on the composites. The term “the dry conditions” refers to condition that the formation of a thin water layer on the composites was not generated.

Table 1 show the experimental conditions and Table 2 show the experimental results of experimental No. 1-7. And, Table 3 show the experimental conditions and Table 4 show the experimental results of experimental No. 8-25. Hence, Tables 1 and 2 make a pair, and, Tables 3 and 4 also make a pair.

**Table 1:** Experimental conditions.

Group	No.	Date	Sample composition	Irradiation time	Weather	Irradiation intensity	Sample condition
			TiO_2_/ZrO_2_			mW/cm^2^	
A	1	2015/7/22	TiO_2_ only	13:30–14:00 (30 min)	Clear	1.65	dry
	2	2015/7/21	TiO_2_ only	11:30–12:00 (30 min)	Clear	1.51	wet
	3	2014/8/3	1/1	14:30–15:00 (30 min)	Clear	1.10	dry
	4	2015/7/23	1/1	14:30–15:00 (30 min)	—	Dark(0)	wet
	5	2017/7/15	noneblank test	11:30–11:35 (5 min)	Clear	1.51	dry
B	6	2017/8/9	1/1mix&press	11:10–11:20 (10 min)	Clear	1.41	wet
	7	2017/8/8	1 + 1no press mix only	12:00–12:10 (10 min)	Clear	1.59	wet

**Table 2:** Experimental results^†^.

Group	No.	Holding in refrigerator	Injector air		Irradiation time	Product/t				Volume of air(after irradiation)^‡^
			Temperature	Humidity		formaldehyde		methanol		
		h	°C	%	tmin	ppm/t	μmole/g·t	ppm/t	μmole/g·t	ml
A	1	0	30.2	64	30	—	—	—	—	1000
	2	20	30.0	67	30	1728	386	279	62	1000
	3	0	31.9	61	30	—	—	—	—	1000
	4	20	29.4	69	30	—	—	300	67	1000
	5	—	29.0	63	5	—	—	—	—	1000
B	6	38	29.6	75	10	2304	514	265	59	1000
	7	22	29.2	76	10	547	122	128	29	1000

^†^Samples of 0.2 g were irradiated with only real solar light. And, sign “−” shows that color change of gas-detecting-tube was not detected (reduced product is equal to almost 0).

^‡^There is inaccuracy from 30 ml to +10 ml.

**Table 3:** Experimental conditions.

Group	No.	Date	Sample composition	Irradiation time	Weather	Irradiation intensi-ty	Sample condition
			TiO_2_/ZrO_2_			mW/cm^2^	
C	8	2016/8/1	TiO_2_ only	10:55–11:00(300 s)	Clear	1.31	wet
	9	2016/8/16	8/2	11:00–11:05(300 s)	Clear	1.36	wet
	10	2016/7/29	8/2	12:55–13:00(300 s)	Clear	1.40	wet
	11	2016/8/17	6/4	11:55–12:00(300 s)	Clear	1.24	wet
	12	2016/9/6	6/4	10:55–11:00(300 s)	Clear	1.16	wet
	13	2016/7/28	4/6	10:55–11:00(300 s)	Clear	1.24	wet
	14	2016/7/29	4/6	11:00–11:05(300 s)	Clear	1.40	wet
	15	2016/7/29	2/8	11:55–12:00(300 s)	Clear	1.49	wet
	16	2016/9/10	ZrO_2_ only	11:00–11:05(300 s)	Clear	1.19	wet
D	17	2015/8/7	1/1	11:55–12:00(300 s)	Thin clouds	0.88	wet
	18	2015/8/2	1/1	11:55–12:00(300 s)	Clear	1.03	wet
	19	2015/8/3	1/1	11:50–11:55(300 s)	Clear	1.06	wet
	20	2015/8/4	1/1	11:55–12:00(300 s)	Thin clouds	0.90	wet
	21	2015/8/5	1/1	11:55–12:00(300 s)	Clear	1.48	wet
	22	2015/8/6	1/1	11:55–12:00(300 s)	Clear	1.33	wet
	23	2015/8/12	1/1	11:58–12:03(300 s)	Clear	1.04	wet
	24	2016/7/20	1/1	14:50–14:55(300 s)	Clouds	0.45	wet
	25	2016/7/29	1/1	14:00–14:05(300 s)	—	Dark(0)	wet

**Table 4:** Experimental results^†^.

Group	No.	Holding in refrigerator	Injector air		Irradiation time	Product/t				Volume of air(after irradiation)^‡^
			Tempe- rature	Humidity		formaldehyde		methanol		
		h	°C	%	t min	ppm/t	μmole/g·t	ppm/t	μmole/g·t	ml
C	8	32	30.2	66	5	259	58	80	18	1000
	9	21	30.7	59	5	374	83	120	27	1000
	10	21	30.3	55	5	432	96	96	21	1000
	11	23	30.6	71	5	432	96	144	32	1000
	12	48	30.4	67	5	518	116	272	61	1000
	13	39	29.1	62	5	315	70	80	18	1000
	14	21	29.7	57	5	288	64	195	44	1000
	15	21	30.0	54	5	135	30	40	9	1000
	16	43	29.8	60	5	20	4	—	—	1000
D	17	20	31.8	61	5	324	72	80	18	1000
	18	20	31.9	64	5	576	129	216	48	1000
	19	20	31.7	62	5	518	116	265	59	1000
	20	20	31.4	66	5	720	161	104	23	1000
	21	20	31.5	67	5	1008	225	265	59	1000
	22	20	31.2	68	5	1152	257	375	84	1000
	23	20	30.4	68	5	585	131	96	21	1000
	24	48	30.1	59	5	115	26	88	20	1000
	25	21	30.4	54	5	—	—	53	12	1000

^†^Samples of 0.2 g were irradiated with only real solar light. And, sign “−” shows that color change of gas-detecting-tube was not detected (reduced product is equal to almost 0).

^‡^There is inaccuracy from 30 ml to +10 ml.

**Figure 3:**
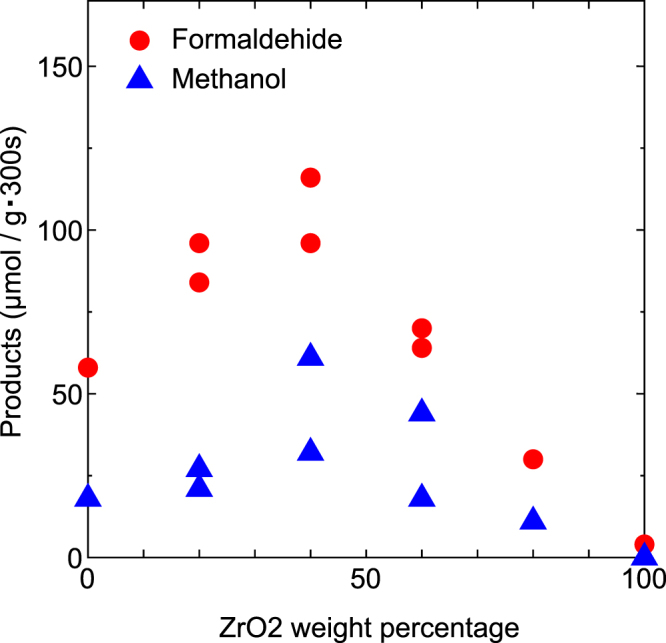
Plots of products against ZrO_2_ weight % in TiO_2_/ZrO_2_ composite. Plots in this figure correspond to the results in the Group C of Tables 3 and 4.
  Moreover, interface of TiO_2_ particle and ZrO_2_ one increases with increasing of ZrO_2_. Increasing of interface increased the reaction products.
  This result agrees with the description of b) in this section
  (d) Group D of Tables 3 and 4
  Group D of Tables 3 and 4 show the results obtained when the composites were used. The results indicate that CO_2_ was reduced under real solar light and that formaldehyde and methanol were obtained in every experiment. A large amount of reduced products (maximum: approximately 300 μmol/(g·300 s) of formaldehyde and methanol)was obtained. These reaction also is catalytic one at the interface of TiO_2_ particle and ZrO_2_ one, because composites were prepared with author’s standard method (mix and press). And, Turnover number (reduction products (mole/h)/catalyst (mole)) was equal to 1.7.
  Figure 4 shows plots of formaldehyde and methanol products (μmol/(g·300 s)) as a function of the irradiation intensity of solar light (mW/cm^2^). These plots reveal that the formaldehyde yield increased rapidly with increasing irradiation energy of solar light. By contrast, the amount of methanol gradually increased with increasing irradiation intensity of solar light. This shows that CO_2_ reducing reaction was enhanced strongly by the photocatalytic effort.

(a) Group A of Tables 1 and 2

- The results in Group A of Tables 1 and 2 show that no reduced product was obtained under dry conditions. By contrast, reduced products were obtained under wet conditions. Therefore, wet conditions are essential.
  Moreover, Group A shows the following results
- Under dry conditions with TiO_2_ only, no products reduced by the photo-catalyst were detected (Tables 1 and 2, No. 1).
  That is, reduction driving force of TiO_2_ photo-catalyst is not enough to reduce the CO_2_.
- Under wet conditions with TiO_2_ only, reduced products were probably obtained by a catalytic- reaction (Tables 1 and 2, No. 2).
- Under dark (no light irradiation) and wet conditions, the TiO_2_/ZrO_2_ composite produced methanol, which is clearly a catalytic- reaction (Tables 1 and 2, No. 4).
- Tables 1 and 2, No. 5 shows result of blank test, that is, formaldehyde and methanol were not generated from the plastic bag under irradiation of solar light.
  (b) Group B of Tables 1 and 2
  On the amount of reduction products, very large deference was showed between No. 6 and No. 7. No. 6 was prepared with strong press during mixing, whereas, No. 7 was prepared with no press (only mixing). On the surface of composites with press during mixing, large amount of interface between TiO_2_ particles and ZrO_2_ ones are formed, whereas, in the case of no press, the interface exist at only contact point between TiO_2_ particles and ZrO_2_ ones. Therefore, possible explanation is that reaction cites exist at the interface. And, this result supports that reaction of this research is catalytic one.
  Incidentally, standard method of this report is strong press during mixing.
  (c) Dependence of quantity of reduced products on TiO_2_/ZrO_2_ ratios
  Figure 3 shows plots of formaldehyde and methanol products (μmol/(g·300 s)) against ZrO_2_ weight % in the TiO_2_/ZrO_2_ composite that correspond to the results in Group C of Tables 3 and 4. Figure 3 shows volcano plots, and, peak was at TiO_2_/ZrO_2_ = 6/4−5/5. In left side of the peak, reduced products increased with increasing of ZrO_2_, and, in right side of the peak, decreased with decreasing of TiO_2_. This shows that activating effect of ZrO_2_ increased in left side of the peak, by contrast, photocatalytic effort of TiO_2_ decreased in right side of the peak. (Band gap of ZrO_2_ is 5.0 eV, whereas, maximum energy of solar light is equal to 4.1 eV (1240/300 nm in wavelength). Band gap of ZrO_2_ is larger than maximum energy of solar light. Therefore, photocatalytic effect of ZrO_2_ does not occur).

**Figure 4:**
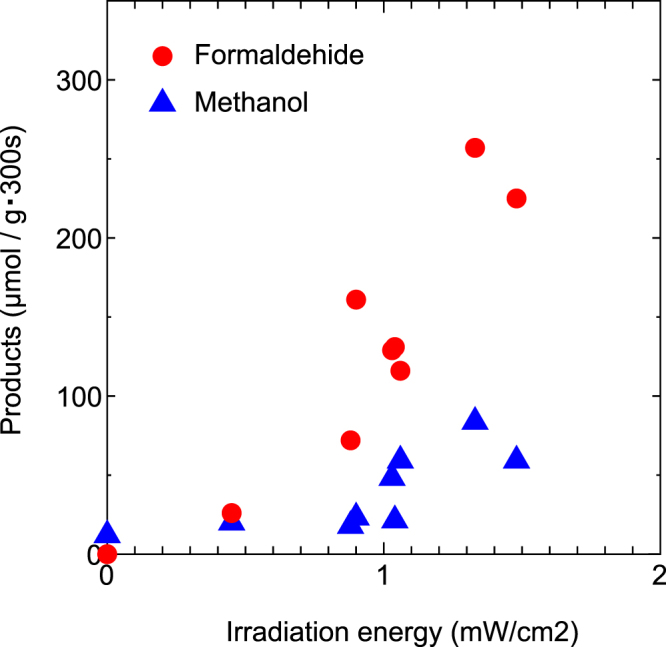
Plots of products as a function of irradiation energy (intensity). The plots in this figure correspond to the results in Group D of Tables 3 and 4.
  (e) Evidence of the generation of the thin water layer
  In this report, thin water layer on the surface of composites is most important.
  As this water layer is very thin, transparent, liquid and not-flat, visualization of water layer was difficult. Thus, below mentioned considerations were performed.
- As described at the end part of the Experiments section, the weight of the test unit increased 70–110 mg, 5–8 min after being removed from the refrigerator. This weight gain certainly includes water-vapour condensation on the surfaces of the composites, the Cu plate and the glass laboratory dish. However, this weight gain also provides indirect evidence of the formation of a thin condensed water layer on the composites because the surface area of the composites is markedly larger than the surface areas of the other materials; i.e., this weight gain would be dominated by the weight gain on the composite surface.
- Electric conductivities of two kinds of composites were measured under dry and wet conditions.

The composites were TiO_2_/ZrO_2_/NaCl (weight ratio) = 4.5/4.5/1 and 4/4/2.

In the case of wet condition, each composite (0.2 g) was scattered in glass laboratory dish, held in the refrigerator for 20–24 h. They were measured in atmosphere 5 min after being removed from refrigerator.

Atmosphere conditions were 26–28 °C in temperature and 73–77% in humidity, respectively. They were measured by HORIBA COMPACT CONDUCTIVITY METER B-771.

The measuring results were

- TiO_2_/ZrO_2_/NaCl = 4.5/4.5/1: conductivity 80 μS (atmosphere: 27.9 °C 73%)
- TiO_2_/ZrO_2_/NaCl = 4.5/4.5/1: conductivity 95 μS (atmosphere: 27.8 °C 74%)
- TiO_2_/ZrO_2_/NaCl = 4/4/2: conductivity 160 μS (atmosphere: 26.1 °C 76%)
- TiO_2_/ZrO_2_/NaCl = 4/4/2: conductivity 250 μS (atmosphere: 27.8 °C 77%)

In contrast to these, in the dry condition, no conductivities of two kinds of composites were detected (electric conductivities were 0 μS).

Therefore, in the case of wet condition, surface of composites were covered with continuous water layer by condensation of water vapour, NaCl dissolved and dissociated in the water layer. Then, the conductivity was generated. This result shows existence of the water layer on the surface of composites.

- If the water layer is not formed, the TiO_2_/ZrO_2_ composite’s surface is dry. As dry TiO_2_ photo-catalyst particles exhibit only strong oxidation activity, no reduced products are produced under UV light irradiation. (Author dispose gas in the bag after the formaldehyde and methanol were returned to CO_2_ by the oxidation effect of dry TiO_2_ particles.) But large amount of reduced products actually generated. This also evidences the existing of water layer on the surface of the composites by the condensation of water vapour.

(f) The role of ZrO_2_

ZrO_2_ have affinity to both acid and base^21^. Further, ZrO_2_ strongly support for hydrogenation of CO and CO_2_ as a carrier^22–25^. Therefore, ZrO_2_ provably have moderate adsorption force with carbonyl group. And, ZrO_2_ probably adsorbs carbonyl group on the surface physically and transport adsorbing species to the interface of TiO_2_ particle and ZrO_2_ one.

It was shown from Fig. 3 that adding of ZrO_2_ largely increased the reduced products. From these results, the use of ZrO_2_ was essential for obtaining large amounts of reduced products.

### The measuring results were

## Discussion

CO_2_ molecules dissolve in the thin layer of water on the composites. Part of the dissolved CO_2_ in the thin water layer generates carbonic acid^26^ (equation (1)):

1 $$C{O}_{2}(aq)+{H}_{2}O={H}_{2}C{O}_{3}$$

The equilibrium constant (K_h_) for this reaction is small (1.7 × 10^−3^ at 25 °C). Furthermore, H_2_CO_3_ dissociates, as described by equations (2) and (3)^27,28^:

2 $${H}_{2}C{O}_{3}(aq)=HC{{O}_{3}}^{-}(aq)+{H}^{+}(aq)\,\,pK{\ast }_{a1}=6.35$$

3 $$HC{O}_{3}^{-}=C{O}_{3}^{2-}(aq)+(aq)\,p{k}_{a2}=10.33$$

where pK*_a1_ and pK_a2_ are the apparent acid-dissociation constant and acid-dissociation constant, respectively.

The reaction formulas that include CO_3_^2−^, HCHO and CH_3_OH are shown as equations (4) and (5); the standard electrode potentials E^0^ are also shown for these equations^29^:

4 $$C{{O}_{3}}^{2-}+6{H}^{+}+4{e}^{-}=HCHO(aq)+2{H}_{2}O\,{E}^{0}(25\,^\circ {\rm{C}})/V=0.197V$$

5 $$C{{O}_{3}}^{2-}+8{H}^{+}+6{e}^{-}=C{H}_{3}OH(aq)+2{H}_{2}O\,\,{E}^{0}(25\,^\circ {\rm{C}})/V=0.209V\,$$

The standard electrode potential E^0^ is related to the standard Gibbs energy transition by following equation:

6 $${E}^{0}=-{\rm{\Delta }}{G}^{0}/nF\,(F:Faraday\,constant)$$

In equation (6), E^0^ is positive; therefore, ∆G^0^ is negative because nF is positive. As ∆G^0^ is negative, the reactions in equations (4) and (5) proceed towards the right to achieve equilibrium if the energy exceeds the activation energy. Catalysts are known to remarkably decrease the activation energy. Thus, equations (4) and (5) through which carbonic ions are reduced are not photocatalytic but catalytic reaction.

CO_3_^2−^ is converted into HCHO and CH_3_OH; then, HCHO and CH_3_OH volatilize from the thin water layer. As the equilibrium (equations (4) and (5)) is disrupted, equations (4) and (5) proceed towards the right to maintain equilibrium, thereby decreasing the concentration of CO_3_^2−^. To maintain the equilibria of equations (1), (2) and (3), CO_2_ dissolves and dissociates and new CO_3_^2−^ is generated. The generation of HCHO and CH_3_OH therefore continues.

These reactions together form a chain reaction, and the reaction from volatilization to the next volatilization is cyclic. Moreover, the thin water layer accelerates this cycle by the facile volatilization of HCHO and CH_3_OH. After the cycle completes, the next cycle continues until the water layer disappears. A large amount of reduced products are obtained. In equation (3), the CO_3_^2−^(aq) concentration is extremely small. However, the aforementioned cyclic reaction can compensate for the low concentration of CO_3_^2−^.

Author calculated vapour pressure (p) of formaldehyde and methanol by Antoine’s formula.

$$\begin{array}{l}\mathrm{Formaldehyde}:\,{\rm{p}}={\rm{2358.0}}\,{\rm{Torr}}\,{\rm{at}}\,{\rm{10}}\,^\circ {\rm{C}}\\ \qquad \quad \,\,\qquad \quad {\rm{p}}={\rm{3284.0}}\,{\rm{Tor\; rat\; 20}}\,^\circ {\rm{C}}\\ \begin{array}{c}\mathrm{Methanol}:\,\,\,\,\quad {\rm{p}}={\rm{55.4}}\,{\rm{Torr}}\,{\rm{at}}\,{\rm{10}}\,^\circ {\rm{C}}\\ \qquad \quad \,\,\qquad \quad {\rm{p}}={\rm{97.3}}\,{\rm{Torr}}\,{\rm{at}}\,{\rm{20}}\,^\circ {\rm{C}}\end{array}\end{array}$$

(Vapour pressure of water: p = 9.2 Torr at 10 °C and 17.5 Torr at 20 °C)^30^

These results show a relation of formaldehyde > methanol > water.

This result (formaldehyde > ethanol) was in agreement with order of the quantity of reduced products (formaldehyde > ethanol).

The solar light that reaches ground is greater than 300 nm in wavelength. The TiO_2_ photo-catalyst absorbs light less than 380 nm in wavelength. Therefore, the available wavelength range is only 300–380 nm. The energy of the light in this range is estimated to be approximately 3–4% of the entire solar energy.

However, solar energy of 3–4% is useful. Both e^−^ and h^+^ are generated by the TiO_2_ photo-catalyst.

In this report, the author used only TiO_2_, not Pt/TiO_2_. Therefore, the reaction schemes are based on the report of Fujishima *et al*.^17^:

$${{\rm{TiO}}}_{2}+2\,\mathrm{photons}(h\upsilon )\to {{\rm{2e}}}^{-}+2{{\rm{h}}}^{+}$$

At oxidation sites on the TiO_2_ surface: H_2_O + 2 h^+^ → 1/2 O_2_ + 2H^+^

At reduction sites on the TiO_2_ surface: 2 e^−^ generate the reduction driving force

H^+^ and e^−^ can be used as those in equation (4) and (5).

Reduction driving force of electron generated by TiO_2_ photo-catalyst is not enough to reduce the CO_2_ directly. However, as the reduction driving force (−0.52 eV with respect to SHE^31,32^) is stronger than that of normal electrons, and, high energy e^−^ ions can move to the interface of TiO_2_ particle and ZrO_2_ one. And, the catalytic reducing reaction can be enhanced by the strong reduction driving force of e^−^ ions at the interface. Figure 4 showed that products yield of formaldehyde and methanol largely increased with increasing irradiation intensity of solar light. Increasing of irradiation intensity offers increasing numbers of high energy electron, thus, catalytic reducing reaction was enhanced.

From results and discussion, possible macro mechanism is following

1. Dissolve and dissociate of CO_2_ in air into the thin water layer on the surface of composite, and generation of CO_3_^2−^ ions in the thin water layer.
2. Physical adsorbing of CO_3_^2−^ ion to the ZrO_2_ surface.
3. Movement of adsorbing species to the interface of TiO_2_ particle and ZrO_2_ one.
4. Catalytic reducing reaction of adsorbing species at the interface.
  High energy e^−^ generated by TiO_2_ photocatalytic effect strongly accelerates the catalytic reducing reaction.
5. Desorption of reduced products (low-molecular-weight organic compounds) from the interface into thin water layer.
6. Evaporation of reduced products (low-molecular-weight organic compounds) from the water thin layer into air.
7. Repeating of 1–6.

## Conclusion

In this study, the thin water layer noted by Sato *et al*. splits to provide H^+^ ions because of the TiO_2_ photo-catalyst and provides a reaction medium for the catalytic reduction of CO_2_.

Thin water layer on the composite surface (wet condition) is essential, and, author’s method is catalytic reaction enhanced by the TiO_2_ photo-catalyst. This method is new approach upon which nobody could hit. Moreover, the fact that a large amount of reduced products was obtained from CO_2_ and H_2_O in air under irradiation of only real solar light at room temperature and atmosphere pressure is a novel and important finding.

## Methods

### Preparation of the composites (c)

In the case of TiO_2_/ZrO_2_ = 1/1, procedure is following.

First, 0.5 g of nanometre-sized anatase TiO_2_ particles (a) (TAYKA Co. Ltd., AMT100, nominal particle size: 6 nm) and 0.5 g of special-grade ZrO_2_ particles (b) (KANTO KAGAKU Co. Ltd., purity: 99.9%, particle size: 10–15 μm) were placed into a small ceramic pot and mixed uniformly. They were then forcefully pressed and agglomerated at room temperature to form the composites (c).

### Experimental procedure

Next, 0.2 g of the composites (c) was scattered uniformly onto a copper plate, which was placed in a glass laboratory dish (inner diameter: 56 mm). The assembly with (c) scattered on the Cu plate in a glass laboratory dish is referred to as the test unit.

Initially, particles or molecules adsorbed onto the composites surface were removed by eliminating static electricity using an ion blower (AZ ONE Co. Ltd., Ion Blower AS-18). The blowing time was 1 h in the strong-wind mode. (Without the use of the ion blower, the test unit must be kept in a refrigerator for 2–3 days).

After the test unit was kept for more than 20 h in a refrigerator (inner temperature of the refrigerator: 1–5 °C), it was removed and placed into a transparent gas-barrier plastic bag (OKURA KOGYO Co. Ltd., OE-4, 200 × 300 mm^2^). The bag was heat-sealed at its entrance. Urethane tape (1 × 1 cm^2^) was adhered to the outside surface of the bag. After the air in the bag was removed using a plastic syringe, new air was injected into the bag. The needle of the syringe was inserted though the urethane tape to avoid leaving a small hole after the needle was removed. The inner volume of the bag was 1000 ml. The bag was then placed on the ground facing the sun.

The injected air was at a sufficient temperature and humidity. With the using of 0.2 g of the composites (c), the desired temperature and humidity were approximately 30 °C and 60–80%, respectively. Too much quantity of water layer by condensation of water vapour considerably decreased the reduced products, for instance, in the case of 95–100% in humidity.

After irradiation by real solar light through the bag for 0.5 h or 10 min or 300 s, the concentrations (ppm) of formaldehyde and methanol were measured by a gas-detecting tube (GASTEC Co. Ltd., tube No. 91 M (measuring range: 8–20 ppm, 20–2000 ppm, and 2000–6400 ppm; three measuring ranges were available by altering the suction volume) and Tube No. 111L (measuring range: 20–40 ppm and 40–1000 ppm; two measuring ranges are available by altering the suction volume)). The tubes were inserted one by one thorough the plastic tape adhered to the outside surface of the bag, and the concentrations were measured; after the tubes were removed, the remaining hole was immediately sealed with another piece of tape. The inner volume of the bag was measured by extracting the gas with the plastic syringe. The measured concentrations (ppm) of both formaldehyde and methanol were then corrected for temperature and expressed in terms of μmol/g:

$$\mathrm{Micromole}/{\rm{g}}=\mathrm{cV}/(\mathrm{22},\mathrm{400}\times {\rm{g}})$$

where, c is the gas concentration (ppm), V is the inner volume after irradiation (ml), and g is the mass of composite (c) used (g).

The intensity of the UV light (mW/cm^2^) with a wavelength of 365 nm was measured using a UNIMETER (USHIO Co. Ltd., UIT-201 and UVD-365PD) during the irradiation of solar light during each experiment; the measurements were performed near the point where the composites were placed in the bag. The intensity was measured every 5 min (Experiment Nos 1–4 and Nos 17–25) or every 1 min (Experiment Nos 5–16). In the case of experiment Nos 17–25, the intensity was measured at the start and the end of irradiation. And, average of each experiment was calculated.
